# Spacecraft Angular Rates Estimation with Gyrowheel Based on Extended High Gain Observer

**DOI:** 10.3390/s16040537

**Published:** 2016-04-14

**Authors:** Xiaokun Liu, Yu Yao, Kemao Ma, Hui Zhao, Fenghua He

**Affiliations:** School of Astronautics, Harbin Institute of Technology, No.2 Yikuang Street, Nangang District, Harbin 150080, China; yaoyu@hit.edu.cn (Y.Y.); makemao@hit.edu.cn (K.M.); zhaohui@hit.edu.cn (H.Z.); hefenghua@hit.edu.cn (F.H.)

**Keywords:** gyrowheel, angular rates estimation, torque output, extended high gain observer, Lyapunov stability, measurement noise

## Abstract

A gyrowheel (GW) is a kind of electronic electric-mechanical servo system, which can be applied to a spacecraft attitude control system (ACS) as both an actuator and a sensor simultaneously. In order to solve the problem of two-dimensional spacecraft angular rate sensing as a GW outputting three-dimensional control torque, this paper proposed a method of an extended high gain observer (EHGO) with the derived GW mathematical model to implement the spacecraft angular rate estimation when the GW rotor is working at large angles. For this purpose, the GW dynamic equation is firstly derived with the second kind Lagrange method, and the relationship between the measurable and unmeasurable variables is built. Then, the EHGO is designed to estimate and calculate spacecraft angular rates with the GW, and the stability of the designed EHGO is proven by the Lyapunov function. Moreover, considering the engineering application, the effect of measurement noise in the tilt angle sensors on the estimation accuracy of the EHGO is analyzed. Finally, the numerical simulation is performed to illustrate the validity of the method proposed in this paper.

## 1. Introduction

The micro-spacecraft is a major trend in the development of space technology, since it has many advantages, such as being light weight, having low power, low cost and high integration [1]. However, when designing the micro-spacecraft, the researchers meet some issues, such as stringent mass, power and volume constraints, which significantly impact cost [2]. The attitude control system (ACS) in a micro-spacecraft, which mainly includes sensors, actuators and some control logics, accounts for a major proportion of the above-mentioned constraints [3]. In order to improve the functional integration destiny in a unit of space and the redundancy of the ACS for the micro-spacecraft, researchers attempt to exploit the fullest potential to integrate the attitude-sensing and attitude-control functions into a single device, which can significantly alleviate the constraints of the cost factors, such as mass, power and volume [4].

Previous research has demonstrated that according to the difference of the gyro rotor support, the integrated devices combining both attitude-sensing and the attitude-control can be roughly divided into two categories: the magnetically-suspended double-gimbal control moment gyroscope (CMG) (MSDGCMG) [4,5,6] and the integrated device-based gimbal support structure represented by a gyrowheel (GW) [7,8]. The former MSDGCMG supports the rotor by active magnetic bearings (AMBs) [9,10]. The latter GW is developed based on the principle of a dynamically-tuned gyroscope (DTG) by Bristol Aerospace Company for the Canadian Space Agency’s SCISAT-1 Scientific in 2003, and the GW rotor is supported by crossed torsion springs and a gimbal. The MSDGCMG implements the functions of the two degrees of freedom (DOF) torque output and two-axis angular rate sensing in two different operation modes [3,11], so that it can only implement a type of function at some point. However, unlike MSDGCMG, the GW not only has the ability for 3-DOF torque output and two-axis angular rate sensing, but also can implement both functions at the same time because of its simple structure.

This paper focuses on the problem of the two-axis angular rate sensing of a GW. Actually, this problem has been partly studied by Canadian academics at Carleton University. For example, in order to implement the two-axis angular rate sensing of the carrier by a GW, Dr. Own in Canada achieved this work by linearizing the motion equations of the GW at the zero tilt angle position and ignoring the effects of the angular acceleration of the carrier and the tilt angle acceleration of the GW rotor [12]. Combining with the static calibration experiments [13], the two-axis angular rates can be measured accurately by this method when the GW rotor works in a smaller rotor tilt angle region (<0.1°). However, it is hard to meet the requirement of the radial torque output for the smaller working tilt angle of the GW rotor. Therefore, in order to implement the radial torque output, the GW rotor frequently works in a larger tilt angle region, which can be up to $7^{\circ }$, and the tilt angles are time-varying, which makes the operation of linearization and the omission of tilt angle acceleration unreasonable. Moreover, the GW is applied to a strapdown inertial navigation system (SINS), which means the GW base is directly fixed on the spacecraft, and according to the gyroscope principle, the complicated angular motion of the spacecraft inevitably affects the dynamic characteristics of GW because of the large moment of inertia and spinning speed of the GW rotor. Therefore, the effect of spacecraft motion on the GW needs to be comprehensively considered. All of the above-mentioned factors result in a larger accuracy loss of angular rate sensing using the above linearized algebra measurement equations.

In this paper, the angular rate sensing problem of the GW is presented when the rotor works in larger tilt angle states. In order to deal with this problem, a novel dynamic estimation approach of spacecraft angular rates based on GW is proposed. This approach provides most of the derived nonlinear mathematical model of the GW built by Lagrange’s method and develops a nonlinear estimation algorithm to implement the angular rate sensing of spacecraft when the GW works in large tilt angles. To this end, the remainder of this paper is divided into four sections: In Section 2, the description of the GW physical structure is firstly presented, then the GW dynamic equations are derived, and the relationships between the measurable variables and unmeasurable variables are built for the subsequent angular rate measurement. In Section 3, the extended high gain observer (EHGO) is designed to implement the estimation of the related terms of the angular rates of the spacecraft, and the error convergence of the designed EHGO is proven in the time domain. In Section 4, the effects on the observer accuracy of the EHGO from measurement noise are further analyzed. In Section 5, for validating the performance of the proposed approach, the numerical simulation is performed. Finally, the conclusions are drawn in Section 6.

## 2. Descriptions of Gyrowheel

### Gyrowheel Physical Structure

**Figure 1:**
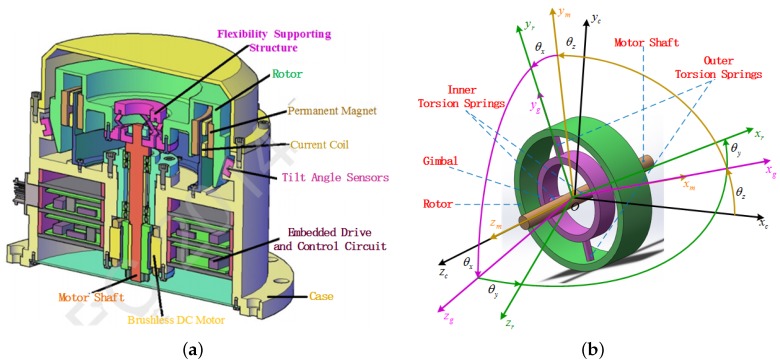
GW physical structure. (**a**) Schematic diagram of a gyrowheel system; (**b**) Simplified gyrowheel structure diagram.

The GW system schematic diagram and simplified structure as shown in Figure 1 are similar to a dynamically-tuned gyroscope (DTG). As in the computer-aided design diagram shown in Figure 1a, the GW system mainly consists of the following subassemblies: case, motor, flexibility suspension structure, flywheel rotor, torquer consisting of the current coil and a permanent magnet and the tilt sensor. Among them, the case is fixed on the carrier, such as a spacecraft. The flexibility suspension structure is made up of a gimbal and inner and outer torsion springs, as shown in Figure 1b; the gimbal is connected to the motor shaft by a pair of inner torsion springs, and the rotor is connected to the gimbal by a pair of outer torsion springs. The rotor driven by the brushless DC motor rotates with a high time-varying speed. Thus, the torque along the spin direction of the rotor can be generated by adjusting the motor speed. Two pairs of torquers perpendicular to each other can provide two-dimensional tilt control torque to make the spin axis of the rotor tilt along the transverse directions. Additionally, the tilt sensors are designed to measure the tilt angles of the rotor with respect to the case. The special physical structure of the GW determines that the device not only can measure the two-dimensional angular rates of the spacecraft, like the DTG, but also can implement the three-dimensional torque output, like the variable speed double gimbal control moment gyroscope (VS-DGCMG).

## 3. Gyrowheel Mathematical Modeling

### 3.1. Gyrowheel Coordinates and Frames

**Figure 2:**
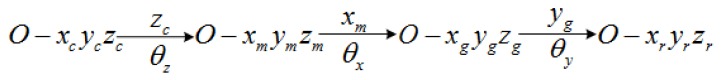
Angular position relationship among the body frames.

The simplified structure and the respective body frames of GW are shown in Figure 1b. The four body frames are case frame($F_{0}$:*O*-$x_{c}y_{c}z_{c}$), motor body frame ($F_{1}$:*O*-$x_{m}y_{m}z_{m}$), gimbal body frame ($F_{2}$:*O*-$x_{g}y_{g}z_{g}$) and rotor body frame ($F_{3}$:*O*-$x_{r}y_{r}z_{r}$), respectively. And Figure 2 shows the angular position relationship of these four body frames. The rotation angles $\theta_{x},\theta_{y},\theta_{z}$ in Figure 1a and Figure 2 are defined as chosen generalized coordinates for GW and can represent the motion about the three degrees of freedom of the GW system. They will be applied to derive the dynamical model of GW using Lagrange’s equations and can be termed as spinning coordinates [2].

According to Figure 2, the direction cosine matrix of rotor with respect to the case can be given by:

(1) $$A={\left(\theta_{y}\right)}_{y}{\left(\theta_{x}\right)}_{x}{\left(\theta_{z}\right)}_{z}=\left(\begin{array}{ccc} C_{\theta_{y}}C_{\theta_{z}}-S_{\theta_{x}}S_{\theta_{y}}S_{\theta_{z}} & C_{\theta_{y}}S_{\theta_{z}}+S_{\theta_{x}}S_{\theta_{y}}C_{\theta_{z}} & -C_{\theta_{x}}S_{\theta_{y}}\\ -C_{\theta_{x}}S_{\theta_{z}} & C_{\theta_{x}}C_{\theta_{z}} & S_{\theta_{x}}\\ S_{\theta_{y}}C_{\theta_{z}}+S_{\theta_{x}}C_{\theta_{y}}S_{\theta_{z}} & S_{\theta_{y}}S_{\theta_{z}}-S_{\theta_{x}}C_{\theta_{y}}C_{\theta_{z}} & C_{\theta_{x}}C_{\theta_{y}} \end{array}\right)$$

where ${\left(\theta_{i}\right)}_{i},i=x,y,z$ represent the rotation matrix of $\theta_{i}$ about the $z_{m}$-axis,$x_{g}$-axis and $y_{g}$-axis, respectively, and $S_{\theta_{i}}=\sin\theta_{i}$, $C_{\theta_{i}}=\cos\theta_{i},i=x,y,z$.

**Figure 3:**
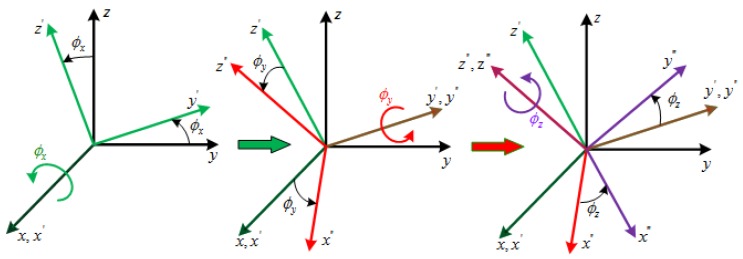
Case-Reference Coordinates and Reference Frames.

Since the two-dimensional tilt sensors of the GW system in Figure 1a can measure the tilt angles of rotor with respect to the case, another set of case-referenced frames $\left(F_{0}^{\prime },F_{1}^{\prime },F_{2}^{\prime },F_{3}^{\prime }\right)$ denoted by the orthogonal triads $\left(x,y,z\right),\left(x^{\prime },y^{\prime },z^{\prime }\right),\left(x^{\prime \prime },y^{\prime \prime },z^{\prime \prime }\right),\left(x^{\prime \prime \prime },y^{\prime \prime \prime },z^{\prime \prime \prime }\right)$ and case-reference coordinates $\left(\phi_{x},\phi_{y},\phi_{z}\right)$, respectively, need to be defined as shown in Figure 3. The frames $F_{0}^{\prime },F_{3}^{\prime }$ are consistent with the frames $F_{0},F_{3}$, and $\phi_{x},\phi_{y}$ can be measured directly by the tilt sensors in Figure 3. According to the rotation order of the case-referenced frames, the direction cosine matrix of rotor with respect to the case can be given by:

(2) $$A^{\prime }={\left(\phi_{z}\right)}_{z}{\left(\phi_{y}\right)}_{y}{\left(\phi_{x}\right)}_{x}=\left[\begin{array}{ccc} C_{\phi_{y}}C_{\phi_{z}} & S_{\phi_{x}}S_{\phi_{y}}C_{\phi_{z}}+C_{\phi_{x}}S_{\phi_{z}} & -C_{\phi_{x}}S_{\phi_{y}}C_{\phi_{z}}+S_{\phi_{x}}S_{\phi_{z}}\\ -C_{\phi_{y}}S_{\phi_{z}} & -S_{\phi_{x}}S_{\phi_{y}}S_{\phi_{z}}+C_{\phi_{x}}C_{\phi_{z}} & C_{\phi_{x}}S_{\phi_{y}}S_{\phi_{z}}+S_{\phi_{x}}C_{\phi_{z}}\\ S_{\phi_{y}} & -S_{\phi_{x}}C_{\phi_{y}} & C_{\phi_{x}}C_{\phi_{y}} \end{array}\right]$$

where ${\left(\phi_{i}\right)}_{i}$, i=x,y,z represent the rotation matrix of $\phi_{i}$ about the *x*-axis,$y^{\prime }$-axis and $z^{\prime \prime }$-axis, respectively, and $S_{\phi_{i}}=\sin\phi_{i}$, $C_{\phi_{i}}=\cos\phi_{i}$, i=x,y,z.

### 3.2. Gyrowheel Kinematic Equations

Supposing the angular velocity of the GW case fixed on the spacecraft, with respect to inertial space in the case frame $F_{0}$, is $\omega_{b}={\left[\begin{array}{ccc} \omega_{bx} & \omega_{by} & \omega_{bz} \end{array}\right]}^{T}$

According to the frames conversion relationship shown in Figure 3, the angular velocity of the motor shaft $\omega_{m}$ in the motor body frame $F_{1}$ is presented as the Equation (3).

(3) $$\omega_{m}=\left[\begin{array}{c} \omega_{mx}\\ \omega_{my}\\ \omega_{mz} \end{array}\right]=\left[\begin{array}{c} 0\\ 0\\ {\dot{\theta }}_{z} \end{array}\right]+{\left(\theta_{z}\right)}_{z}\cdot \omega_{b}=\left[\begin{array}{c} \omega_{bx}C_{\theta_{z}}+\omega_{by}S_{\theta_{z}}\\ -\omega_{bx}S_{\theta_{z}}+\omega_{by}C_{\theta_{z}}\\ {\dot{\theta }}_{z}+\omega_{bz} \end{array}\right]$$

The gimbal angular velocity $\omega_{g}$ in its body frame $F_{2}$ is a function of the base and motor shaft angular velocities where:

(4) $$\omega_{g}=\left[\begin{array}{c} \omega_{gx}\\ \omega_{gy}\\ \omega_{gz} \end{array}\right]=\left[\begin{array}{c} {\dot{\theta }}_{x}\\ 0\\ 0 \end{array}\right]+{\left(\theta_{x}\right)}_{x}\cdot \omega_{m}=\left[\begin{array}{c} {\dot{\theta }}_{x}+\omega_{bx}C_{\theta_{z}}+\omega_{by}S_{\theta_{z}}\\ -\omega_{bx}C_{\theta_{x}}S_{\theta_{z}}+\omega_{by}C_{\theta_{x}}C_{\theta_{z}}+\left({\dot{\theta }}_{z}+\omega_{bz}\right)S_{\theta_{x}}\\ \omega_{bx}S_{\theta_{x}}S_{\theta_{z}}-\omega_{by}S_{\theta_{x}}C_{\theta_{z}}+\left({\dot{\theta }}_{z}+\omega_{bz}\right)C_{\theta_{x}} \end{array}\right]$$

Finally, the rotor angular velocity $\omega_{r}$ in its body frame $F_{3}$ is calculated by the gimbal angular velocity and the rotation of the rotor about the outer torsion shaft where:

(5) $$\begin{array}{cc} \omega_{r} & =\left[\begin{array}{c} \omega_{rx}\\ \omega_{ry}\\ \omega_{rz} \end{array}\right]=\left[\begin{array}{c} 0\\ {\dot{\theta }}_{y}\\ 0 \end{array}\right]+{\left(\theta_{y}\right)}_{y}\cdot \omega_{g}\\ & =\left[\begin{array}{c} {\dot{\theta }}_{x}C_{\theta_{y}}-{\dot{\theta }}_{z}C_{\theta_{x}}S_{\theta_{y}}-\omega_{bz}C_{\theta_{x}}S_{\theta_{y}}+\left(C_{\theta_{y}}C_{\theta_{z}}-S_{\theta_{x}}S_{\theta_{y}}S_{\theta_{z}}\right)\omega_{bx}+\left(C_{\theta_{y}}S_{\theta_{z}}+S_{\theta_{x}}S_{\theta_{y}}C_{\theta_{z}}\right)\omega_{by}\\ {\dot{\theta }}_{z}S_{\theta_{x}}+{\dot{\theta }}_{y}-C_{\theta_{x}}S_{\theta_{z}}\omega_{bx}+C_{\theta_{x}}C_{\theta_{z}}\omega_{by}+\omega_{bz}S_{\theta_{x}}\\ {\dot{\theta }}_{x}S_{\theta_{y}}+{\dot{\theta }}_{z}C_{\theta_{x}}C_{\theta_{y}}+\omega_{bz}C_{\theta_{x}}C_{\theta_{y}}+\left(S_{\theta_{y}}C_{\theta_{z}}+S_{\theta_{x}}C_{\theta_{y}}S_{\theta_{z}}\right)\omega_{bx}+\left(S_{\theta_{y}}S_{\theta_{z}}-S_{\theta_{x}}C_{\theta_{y}}C_{\theta_{z}}\right)\omega_{by} \end{array}\right] \end{array}$$

Ignoring the GW case angular velocity $\omega_{b}$, the Equation (5) can be simplified to:

(6) $$\omega_{r}=\left[\begin{array}{c} {\dot{\theta }}_{x}C_{\theta_{y}}-{\dot{\theta }}_{z}C_{\theta_{x}}S_{\theta_{y}}\\ {\dot{\theta }}_{z}S_{\theta_{x}}+{\dot{\theta }}_{y}\\ {\dot{\theta }}_{x}S_{\theta_{y}}+{\dot{\theta }}_{z}C_{\theta_{x}}C_{\theta_{y}} \end{array}\right]$$

Similarly, without considering the case angular velocity $\omega_{b}$, the rotor angular velocity expressed by case-referenced coordinates $\left(\phi_{x},\phi_{y},\phi_{z}\right)$ in its body frame $F_{3}$ can be given by:

(7) $$\omega_{r}^{\prime }=\left[\begin{array}{c} C_{\phi_{y}}C_{\phi_{z}}\cdot {\dot{\phi }}_{x}+S_{\phi_{z}}\cdot {\dot{\phi }}_{y}\\ -C_{\phi_{y}}S_{\phi_{z}}\cdot {\dot{\phi }}_{x}+C_{\phi_{z}}\cdot {\dot{\phi }}_{y}\\ {\dot{\phi }}_{z}+S_{\phi_{z}}\cdot {\dot{\phi }}_{x} \end{array}\right]$$

### 3.3. Gyrowheel Dynamic Equations

Supposing the principal axes of the body frames $F_{1}$, $F_{2}$, $F_{3}$ are consistent with the inertial principal axis of the motor, gimbal and rotor, respectively. Therefore, we can represent the moments of inertia of the motor, gimbal and rotor as follows.

(8) $$\begin{array}{c} I_{m}=diag\left(I_{mx},I_{my},I_{mz}\right)\\ I_{g}=diag\left(I_{gx},I_{gy},I_{gz}\right)\\ I_{r}=diag\left(I_{rx},I_{ry},I_{rz}\right) \end{array}$$

The GW kinetic energy *T* consists of the kinetic energy of motor shaft, gimbal and rotor, which can be expressed by generalized rotation speed quadratic forms:

(9) $$T=\frac{1}{2}\left(\sum_{i=x,y,z}I_{mi}\omega_{mi}^{2}+\sum_{i=x,y,z}I_{gi}\omega_{gi}^{2}+\sum_{i=x,y,z}I_{ri}\omega_{ri}^{2}\right)$$

The GW potential energy *V* is the sum of the potential energy of the inner and outer torsion deformation, which can be given by:

(10) $$V=(k_{x}\theta_{x}^{2}+k_{y}\theta_{y}^{2})$$

where $k_{x},k_{y}$ are stiffness coefficients of the inner and outer torsion springs, respectively.

Above all, the Lagrange energy function *L* can be defined by:

(11) $$L=T-V=\frac{1}{2}\left(\sum_{i=x,y,z}I_{mi}\omega_{mi}^{2}+\sum_{i=x,y,z}I_{gi}\omega_{gi}^{2}+\sum_{i=x,y,z}I_{ri}\omega_{ri}^{2}\right)-\left(k_{x}\theta_{x}^{2}+k_{y}\theta_{y}^{2}\right)$$

For the GW, Lagrange’s equations are given by:

(12) $$\begin{array}{cc} \frac{d}{dt}\left(\frac{\partial L}{\partial {\dot{\theta }}_{x}}\right)-\frac{\partial L}{\partial \theta_{x}} & =T_{gx}-2c_{x}{\dot{\theta }}_{x} \end{array}$$

(13) $$\begin{array}{cc} \frac{d}{dt}\left(\frac{\partial L}{\partial {\dot{\theta }}_{y}}\right)-\frac{\partial L}{\partial \theta_{y}} & =T_{gy}-2c_{y}{\dot{\theta }}_{y} \end{array}$$

(14) $$\begin{array}{cc} \frac{d}{dt}\left(\frac{\partial L}{\partial {\dot{\theta }}_{z}}\right)-\frac{\partial L}{\partial \theta_{z}} & =T_{gz} \end{array}$$

where $T_{gx},T_{gy},T_{gz}$ are generalized control torques corresponding to the generalized coordinates $\theta_{x},\theta_{y},\theta_{z}$. $c_{x},c_{y}$ are damping coefficients of the inner and outer torsion shaft, respectively.

We assume that the angular motion of the rotor along its spin axis is decoupled from the transverse axes motion, and the effect of the z-axis carrier angular rate $\omega_{bz}$ on the transverse axes motion is ignorable. Thus considering these factors and calculating the first two equations of the Equation (14), we obtain

(15) $$\begin{array}{c} I_{1}\cdot {\ddot{\theta }}_{x}=-c_{x}{\dot{\theta }}_{x}-k_{x}\theta_{x}-\frac{1}{2}I_{2}S_{2\theta_{x}}\cdot {{\dot{\theta }}_{z}}^{2}-I_{3}S_{2\theta_{y}}\cdot {\dot{\theta }}_{x}{\dot{\theta }}_{y}-\left(I_{3}C_{2\theta_{y}}-I_{ry}\right)C_{\theta_{x}}\cdot {\dot{\theta }}_{y}{\dot{\theta }}_{z}+T_{gx}+\\ \;B_{1}\left(\theta \right)\omega_{bx}+B_{2}\left(\theta \right)\omega_{by}+B_{3}\left(\theta \right){\dot{\omega }}_{bx}+B_{4}\left(\theta \right){\dot{\omega }}_{by}+B_{5}\left(\theta \right)\omega_{bx}^{2}+B_{6}\left(\theta \right)\omega_{by}^{2}+B_{7}\left(\theta \right)\omega_{by}^{2}\\ I_{ry}\cdot {\ddot{\theta }}_{y}=-c_{y}{\dot{\theta }}_{y}-k_{y}\theta_{y}-\frac{1}{2}I_{3}C_{\theta_{x}}^{2}S_{2\theta_{y}}\cdot {\dot{\theta }}_{z}^{2}+\frac{1}{2}I_{3}S_{2\theta_{y}}\cdot {\dot{\theta }}_{x}^{2}+\left(I_{3}C_{2\theta_{y}}-I_{ry}\right)C_{\theta_{x}}\cdot {\dot{\theta }}_{x}{\dot{\theta }}_{z}+T_{gy}+\\ \;D_{1}\left(\theta \right)\omega_{bx}+D_{2}\left(\theta \right)\omega_{by}+D_{3}\left(\theta \right){\dot{\omega }}_{bx}+D_{4}\left(\theta \right){\dot{\omega }}_{by}+D_{5}\left(\theta \right)\omega_{bx}^{2}+D_{6}\left(\theta \right)\omega_{by}^{2}+D_{7}\left(\theta \right)\omega_{by}^{2} \end{array}$$

where $I_{1}=I_{gx}+I_{rx}C_{\theta_{y}}^{2}+I_{rz}S_{\theta_{y}}^{2},I_{2}=I_{gz}-I_{gy}-I_{ry}+I_{rx}S_{\theta_{y}}^{2}+I_{rz}C_{\theta_{y}}^{2},I_{3}=I_{rz}-I_{rx}$ and $B_{i}\left(\theta \right),D_{i}\left(\theta \right)i=1,2\cdots 7$ in Equations (16) and (17) are nonlinear coefficient terms of the spacecraft angular rates($\omega_{bx},\omega_{by}$), respectively.

(16) $$\begin{array}{cc} B_{1}\left(\theta \right)= & \left[-I_{3}S_{\theta x}S_{2\theta_{y}}C_{\theta_{z}}+I_{1}S_{\theta_{z}}+I_{2}\cos2x_{1}S_{\theta_{z}}\right]{\dot{\theta }}_{z}-\left[I_{3}\left(S_{2\theta_{y}}C_{\theta_{z}}+S_{\theta x}C_{2\theta_{y}}S_{\theta_{z}}\right)-I_{ry}S_{\theta x}S_{\theta_{z}}\right]x_{4}\\ B_{2}\left(\theta \right)= & -\left[I_{3}S_{\theta x}S_{2\theta_{y}}S_{\theta_{z}}+I_{1}C_{\theta_{z}}+I_{2}C_{2\theta_{y}}C_{\theta_{z}}\right]{\dot{\theta }}_{z}-\left[I_{3}\left(S_{2\theta_{y}}S_{\theta_{z}}-S_{\theta x}C_{2\theta_{y}}C_{\theta_{z}}\right)+I_{ry}S_{\theta x}C_{\theta_{z}}\right]x_{4}\\ B_{3}\left(\theta \right)= & -I_{1}C_{\theta_{z}}-\frac{1}{2}I_{3}S_{\theta x}S_{2\theta_{y}}S_{\theta_{z}}\;B_{4}\left(\theta \right)=-I_{1}S_{\theta_{z}}+\frac{1}{2}I_{3}S_{\theta x}S_{2\theta_{y}}C_{\theta_{z}}\\ B_{5}\left(\theta \right)= & \frac{1}{2}I_{2}S_{\theta_{z}}^{2}S_{2\theta_{x}}+\frac{1}{4}I_{3}C_{\theta x}S_{2\theta_{y}}S_{2\theta_{z}}\;B_{6}\left(\theta \right)=\frac{1}{2}I_{2}C_{\theta_{z}}^{2}S_{2\theta_{x}}-\frac{1}{4}I_{3}C_{\theta x}S_{2\theta_{y}}S_{2\theta_{z}}\\ B_{7}\left(\theta \right)= & -\frac{1}{2}I_{2}S_{2\theta_{z}}S_{2\theta_{x}}-\frac{1}{2}I_{3}C_{\theta x}S_{2\theta_{y}}C_{2\theta_{z}} \end{array}$$

(17) $$\begin{array}{cc} D_{1}\left(\theta \right)= & \left[I_{3}\left(S_{2\theta_{y}}C_{\theta_{z}}+S_{\theta_{x}}C_{2\theta_{y}}S_{\theta_{z}}\right)-I_{ry}S_{\theta_{x}}S_{\theta_{z}}\right]{\dot{\theta }}_{x}+\left[I_{3}\left(C_{\theta_{x}}C_{2\theta_{y}}C_{\theta_{z}}-\frac{1}{2}S_{2\theta_{x}}S_{2\theta_{y}}S_{\theta_{z}}\right)+I_{ry}C_{\theta_{x}}C_{\theta_{z}}\right]{\dot{\theta }}_{z}\\ D_{2}\left(\theta \right)= & \left[I_{3}\left(S_{2\theta_{y}}S_{\theta_{z}}-S_{\theta_{x}}C_{2\theta_{y}}C_{\theta_{z}}\right)+I_{ry}S_{\theta_{x}}C_{\theta_{z}}\right]{\dot{\theta }}_{x}+\left[I_{3}\left(\frac{1}{2}S_{2\theta_{x}}S_{2\theta_{y}}C_{\theta_{z}}+C_{\theta_{x}}C_{2\theta_{y}}S_{\theta_{z}}\right)+I_{ry}C_{\theta_{x}}S_{\theta_{z}}\right]{\dot{\theta }}_{z}\\ D_{3}\left(\theta \right)= & I_{ry}C_{\theta_{x}}S_{\theta_{z}}\;D_{4}\left(\theta \right)=-I_{ry}C_{\theta_{x}}C_{\theta_{z}}\;D_{5}\left(\theta \right)=+\frac{1}{2}I_{3}\left[S_{\theta_{x}}C_{2\theta_{y}}S_{2\theta_{z}}+S_{2\theta_{y}}\left(C_{\theta_{z}}^{2}-S_{\theta_{x}}^{2}S_{\theta_{z}}^{2}\right)\right]\\ D_{6}\left(\theta \right)= & -\frac{1}{2}I_{3}\left[S_{\theta_{x}}C_{2\theta_{y}}S_{2\theta_{z}}-S_{2\theta_{y}}\left(S_{\theta_{z}}^{2}-S_{\theta_{x}}^{2}C_{\theta_{z}}^{2}\right)\right]\\ D_{7}\left(\theta \right)= & +\frac{1}{2}I_{3}\left(S_{\theta_{x}}^{2}S_{2\theta_{y}}S_{2\theta_{z}}-2S_{\theta_{x}}C_{2\theta_{y}}C_{2\theta_{z}}+S_{2\theta_{y}}S_{2\theta_{z}}\right) \end{array}$$

### 3.4. Relationship between the Unmeasurable Variables and the Measurable Variables

According to the GW description in Section 2.1, the two-dimensional tilt angles($\phi_{x},\phi_{y}$) can be measured by tilt sensors, the control torques ($T_{cx},T_{cy}$) can be obtained by measuring the torquer coils currents, the motor speed (${\dot{\theta }}_{z}$) and rotation angles ($\theta_{z}$) can be measured by the Hall sensors or rotary transformers, respectively. However, the variables $T_{gx},T_{gy},\theta_{x},\theta_{y}$ and its derivatives ${\dot{\theta }}_{x},{\dot{\theta }}_{y},{\ddot{\theta }}_{x},{\ddot{\theta }}_{y}$ in Equation (15) are not measurable, so the relationship between the unmeasurable variables and the measurable variables should be built, which means the generalized coordinates ($\theta_{x},\theta_{y}$ and its derivatives) in the GW Equation (15) should be expressed by the case-reference coordinates $\phi_{x},\phi_{y}$, and the torque terms ($T_{gx},T_{gy}$) in Equation (15) should be expressed by the measurable control torques $T_{cx},T_{cx}$. For this purpose, according to the rotation motion characteristics of motor shaft, gimbal and rotor, the relationship between $\left(T_{cx},T_{cy}\right)$ and $\left(T_{gx},T_{gy}\right)$ can be given by:

(18) $$\begin{array}{c} T_{gx}=C_{\theta_{z}}\cdot T_{cx}+S_{\theta_{z}}\cdot T_{cy}\\ T_{gy}=-S_{\theta_{z}}C_{\theta_{x}}\cdot T_{cx}+C_{\theta_{z}}C_{\theta_{x}}\cdot T_{cy} \end{array}$$

Considering that both A in Equation (1) and $A^{\prime }$ in Equation (2) can be used to describe the rotation direction of the rotor with respect to the case, thus the Equation (19) is established as follows:

(19) $$A=A^{\prime }$$

Let $\frac{A_{21}}{A_{22}}=\frac{A_{21}^{\prime }}{A_{22}^{\prime }}$,the relationship between $\phi_{z}$ and the measurable variables $\left(\phi_{x},\phi_{y},\theta_{z}\right)$ can be shown as:

(20) $$\tan\phi_{z}=\frac{C_{\phi_{x}}S_{\theta_{z}}}{C_{\phi_{y}}C_{\theta_{z}}+S_{\phi_{x}}S_{\phi_{y}}S_{\theta_{z}}}$$

To build the relationship between ($\theta_{x},\theta_{y}$) and ($\phi_{x},\phi_{y}$), we take the equation:

$$C_{\theta_{z}}A_{31}+S_{\theta_{z}}A_{32}=C_{\theta_{z}}A_{31}^{\prime }+S_{\theta_{z}}A_{32}^{\prime }$$

and it gives:

(21) $$\begin{array}{c} S_{\theta_{x}}=C_{\phi_{x}}S_{\phi_{y}}S_{\phi_{z}}+S_{\phi_{x}}C_{\phi_{z}}\\ S_{\theta_{y}}=S_{\phi_{y}}C_{\theta_{z}}-S_{\phi_{x}}C_{\phi_{y}}S_{\theta_{z}} \end{array}$$

In addition, since both $\omega_{r}$ in Equation (6) and $\omega_{r}^{\prime }$ in Equation (7) represent the rotor angular velocity in the rotor body frame $F_{3}$ with different coordinates, the following Equation (22) is given by:

(22) $$\omega_{r}=\omega_{r}^{\prime }$$

Rearranging the Equations (6), (7) and (22), (${\dot{\theta }}_{x},{\dot{\theta }}_{y}$) and (${\dot{\phi }}_{x},{\dot{\phi }}_{y},{\dot{\phi }}_{z}$) can be expressed by the following forms:

(23) $$\begin{array}{c} {\dot{\theta }}_{x}=\frac{1}{C_{\theta_{y}}}\left(C_{\phi_{y}}C_{\phi_{z}}{\dot{\phi }}_{x}+S_{\phi_{z}}{\dot{\phi }}_{y}+C_{\theta_{x}}S_{\theta_{y}}{\dot{\theta }}_{z}\right)\\ {\dot{\theta }}_{y}=C_{\phi_{z}}{\dot{\phi }}_{y}-C_{\phi_{y}}S_{\phi_{z}}{\dot{\phi }}_{x}-S_{\theta_{x}}{\dot{\theta }}_{z} \end{array}$$

(24) $$\begin{array}{c} {\dot{\phi }}_{x}=\frac{1}{C_{\phi_{y}}}\left[C_{\phi_{z}}C_{\theta_{y}}{\dot{\theta }}_{x}-S_{\phi_{z}}{\dot{\theta }}_{y}-\left(C_{\theta_{x}}S_{\theta_{y}}C_{\phi_{z}}+S_{\theta_{x}}S_{\phi_{z}}\right){\dot{\theta }}_{z}\right]\\ {\dot{\phi }}_{y}=C_{\theta_{y}}S_{\phi_{z}}{\dot{\theta }}_{x}+C_{\phi_{z}}{\dot{\theta }}_{y}-\left(C_{\theta_{x}}S_{\theta_{y}}S_{\phi_{z}}-S_{\theta_{x}}C_{\phi_{z}}\right){\dot{\theta }}_{z} \end{array}$$

(25) $${\dot{\phi }}_{z}=S_{\theta_{y}}{\dot{\theta }}_{x}+C_{\theta_{x}}C_{\theta_{y}}{\dot{\theta }}_{z}-S_{\phi_{y}}{\dot{\phi }}_{x}$$

Taking the derivatives of the two equations in Equation (24), it yields the following Equation (26):

(26) $$\begin{array}{c} {\ddot{\phi }}_{x}=\frac{{\dot{\phi }}_{y}S_{\phi_{y}}}{C_{\phi_{y}}^{2}}\left[C_{\phi_{z}}C_{\theta_{y}}{\dot{\theta }}_{x}-S_{\phi_{z}}{\dot{\theta }}_{y}-\left(C_{\theta_{x}}S_{\theta_{y}}C_{\phi_{z}}+S_{\theta_{x}}S_{\phi_{z}}\right){\dot{\theta }}_{z}\right]+\\ \;\frac{1}{C_{\phi_{y}}}\left[\begin{array}{c} \left(-{\dot{\phi }}_{z}S_{\phi_{z}}C_{\theta_{y}}-{\dot{\theta }}_{y}C_{\phi_{z}}S_{\theta_{y}}\right){\dot{\theta }}_{x}-{\dot{\phi }}_{z}C_{\phi_{z}}{\dot{\theta }}_{y}+C_{\phi_{z}}C_{\theta_{y}}{\ddot{\theta }}_{x}-S_{\phi_{z}}{\ddot{\theta }}_{y}-\left(C_{\theta_{x}}S_{\theta_{y}}C_{\phi_{z}}+S_{\theta_{x}}S_{\phi_{z}}\right){\ddot{\theta }}_{z}\\ +\left({\dot{\phi }}_{z}C_{\theta_{x}}S_{\theta_{y}}S_{\phi_{z}}-C_{\phi_{z}}\left({\dot{\theta }}_{y}C_{\theta_{x}}C_{\theta_{y}}-{\dot{\theta }}_{x}S_{\theta_{x}}S_{\theta_{y}}\right)-{\dot{\phi }}_{z}S_{\theta_{x}}C_{\phi_{z}}-C_{\theta_{x}}S_{\phi_{z}}{\dot{\theta }}_{x}\right){\dot{\theta }}_{z} \end{array}\right]\\ {\ddot{\phi }}_{y}=\left({\dot{\phi }}_{z}C_{\theta_{y}}C_{\phi_{z}}-{\dot{\theta }}_{y}S_{\theta_{y}}S_{\phi_{z}}\right){\dot{\theta }}_{x}+C_{\theta_{y}}S_{\phi_{z}}{\ddot{\theta }}_{x}+C_{\phi_{z}}{\ddot{\theta }}_{y}-S_{\phi_{z}}{\dot{\phi }}_{z}{\dot{\theta }}_{y}-\left(C_{\theta_{x}}S_{\theta_{y}}S_{\phi_{z}}-S_{\theta_{x}}C_{\phi_{z}}\right){\ddot{\theta }}_{z}-\\ \;\left[{\dot{\phi }}_{z}C_{\theta_{x}}S_{\theta_{y}}C_{\phi_{z}}+S_{\phi_{z}}\left({\dot{\theta }}_{y}C_{\theta_{x}}C_{\theta_{y}}-{\dot{\theta }}_{x}S_{\theta_{x}}S_{\theta_{y}}\right)-\left({\dot{\theta }}_{x}C_{\theta_{x}}C_{\phi_{z}}-{\dot{\phi }}_{z}S_{\theta_{x}}S_{\phi_{z}}\right)\right]{\dot{\theta }}_{z} \end{array}$$

Now the relationship between the unmeasurable variables $\left(\theta_{x},\theta_{y},{\dot{\theta }}_{x},{\dot{\theta }}_{y},{\ddot{\theta }}_{x},{\ddot{\theta }}_{y},T_{gx},T_{gy}\right)$ and the measurable variables (tilt angles $\phi_{x},\phi_{y}$,motor speed ${\dot{\theta }}_{z}$ and motor rotation angle $\theta_{z}$) can be expressed by Equations (15), (18), (20), (21), (23) and (25).

### 3.5. Description of Angular Rates Estimation Problem Using Gyrowheel State Equation

Applying the Equations (15), (18), (20), (21), (23) and (25) to the Equation (26), and choosing the measurable tilt angles ($\phi_{x},\phi_{y}$) and its derivatives as the state variables:

$$x={\left[\begin{array}{cccc} x_{1} & x_{2} & x_{3} & x_{4} \end{array}\right]}^{T}={\left[\begin{array}{cccc} \phi_{x} & {\dot{\phi }}_{x} & \phi_{y} & {\dot{\phi }}_{y} \end{array}\right]}^{T}$$

GW state equation can be constructed in terms of x as Equation (27):

(27) $$\begin{array}{c} \left\{\begin{array}{c} {\dot{x}}_{1}=x_{2}\\ {\dot{x}}_{2}=f_{1}\left(x,t\right)+w_{1}\left(\omega_{b},x,t\right)+g_{x}^{1}\left(x,t\right)u_{x}+g_{y}^{1}\left(x,t\right)u_{y}\\ {\dot{x}}_{3}=x_{4}\\ {\dot{x}}_{4}=f_{2}\left(x,t\right)+w_{2}\left(\omega_{b},x,t\right)+g_{x}^{2}\left(x,t\right)u_{x}+g_{y}^{2}\left(x,t\right)u_{y} \end{array}\right. \end{array}$$

Considering that the tilt angles ($\phi_{x},\phi_{y}$) can be measured by the tilt sensors, measurement equation of GW can be shown as:

(28) y=Cx

Further more, GW dynamical equations can be rewritten by the following forms:

(29) $$\left\{\begin{array}{c} \dot{x}=\mathrm{Ax}+B\left[f\left(x,t\right)+w\left(\omega_{b},x,t\right)+g\left(x,t\right)u\right]\\ y=\mathrm{Cx} \end{array}\right.$$

where

$$\begin{array}{c} A=\left[\begin{array}{cccc} 0 & 1 & 0 & 0\\ 0 & 0 & 0 & 0\\ 0 & 0 & 0 & 1\\ 0 & 0 & 0 & 0 \end{array}\right]\;B=\left[\begin{array}{cc} 0 & 0\\ 1 & 0\\ 0 & 0\\ 0 & 1 \end{array}\right]\;C=\left[\begin{array}{cccc} 1 & 0 & 0 & 0\\ 0 & 0 & 1 & 0 \end{array}\right]\;u=\left[\begin{array}{c} u_{x}\\ u_{y} \end{array}\right]=\left[\begin{array}{c} T_{cx}\\ T_{cy} \end{array}\right]\\ f\left(x,t\right)=\left[\begin{array}{c} f_{1}\left(x,t\right)\\ f_{2}\left(x,t\right) \end{array}\right]\;w\left(\omega_{b},x,t\right)=\left[\begin{array}{c} w_{1}\left(\omega_{b},x,t\right)\\ w_{2}\left(\omega_{b},x,t\right) \end{array}\right]\;g\left(x,t\right)=\left[\begin{array}{cc} g_{x}^{1}\left(x,t\right) & g_{y}^{1}\left(x,t\right)\\ g_{x}^{2}\left(x,t\right) & g_{y}^{2}\left(x,t\right) \end{array}\right] \end{array}$$

f(x,t) is a vector irrelevant to the spacecraft angular rates $\omega_{b}$, and tilt control torque u, $w\left(\omega_{b},x,t\right)$ is a vector consisting of the related terms of spacecraft angular rates $\omega_{b}$, u is the tilt control torque vector. Specifically, $f_{i}\left(x,t\right),w_{i}\left(x,t\right)$ and $g_{j}^{i}\left(x,t\right),i=1,2,j=x,y$ can be obtained by combining the simultaneous Equations (15), (20), (21), (23), (25) and (26) with the physical significance of each term, and these concrete expressions are shown in Appendix A. $u_{x}=T_{cx}=k_{ty}i_{y},u_{y}=T_{cy}=k_{tx}i_{x}$, $k_{tx},k_{ty}$ are scale factors of torquers along x-axis and y-axis, $i_{x},i_{y}$ are measurable currents of torquer coils, respectively. In the following of this paper, the moments of inertial of both rotor and gimbal along transverse axis and spin axis are supposed as:

$$I_{rx}=I_{ry}=I_{rt}\;I_{rz}=I_{rs}\;I_{gx}=I_{gy}=I_{gt}\;I_{gz}=I_{gs}$$

For the GW physical system carried on the spacecraft, since the GW torquers and motor power are limited and the spacecraft bandwidth is small, the tilt angles of the rotor, the rotation speed of the motor and the motion of the spacecraft are always continuous and bounded in Equation (29), so the following assumptions hold:

**Assumption 1**:  both the control torques u(x,t) and spacecraft angular rates $\omega_{b}$ are bounded input signals, and spacecraft angular rates ($\omega_{bx},\omega_{by}$) are derivable and their derivatives are bounded.

In addition, since the state x represents the tilt angles and tilt angular rates of the rotor along the transverse axis in the case frame $F_{0}$, the tilt angle range is usually limited to $\left[-5^{\circ },+5^{\circ }\right]$ by mechanical stoppages, then the Assumption 2 is given by:

**Assumption 2**:  The state x is uniformly continuously bounded.

Another main objective of this paper is to design a high gain observer(HGO) featuring global asymptotic convergence to estimate the nonlinear related terms $w\left(\omega_{b},x,t\right)$ about the angular rates of the spacecraft, then the spacecraft angular rates ($\omega_{bx},\omega_{by}$) can be calculated by solving the differential equations containing the angular rates ($\omega_{bx},\omega_{by}$) with the direct estimates.

Through the following study of this paper, these four problems of spacecraft angular rates estimation with GW will be dealt with:

(1) The errors caused by the linearization of nonlinear equations of GW in large tilt angles can be avoided.
(2) The problem of dynamic drift error compensation existing both traditional mechanic gyroscopes [14] and GW can be solved by the above derivation and the following application of full dynamical model in angular rates estimation.
(3) The initial iteration error accumulation of the calculated angular rates of the spacecraft, caused by the angular acceleration (${\dot{\omega }}_{bx},{\dot{\omega }}_{by}$) in the term $w(\omega_{b},x,t)$, is eliminated by the real-time estimation of the $w(\omega_{b},x,t)$ term.
(4) The amplification of measurement noise by the multi-difference of measured tilt angles with measurement noise can be weakened by appropriately choosing the design parameters of HGO [15].

It is noted that the system parameters such as $I_{rt},I_{gt},I_{rs},I_{gs},c_{x},c_{y},k_{x},k_{y},k_{tx},k_{ty}$ in Equation (29) can be identified by the calibration experiments both on-orbit and on-ground.

## 4. Gyrowheel High Gain Observer for Angular Rates Estimation

### 4.1. Gyrowheel High Gain Observer Design

To implement the estimation of the state x and the spacecraft angular rates related term $w\left(\omega_{b},x,t\right)$ using the measurable states ($x_{1},x_{3}$) by tilt angle sensors, we need to extend the existing states x and design the following extended high gain observer:

(30) $$\left\{\begin{array}{c} \dot{\hat{x}}=A\hat{x}+B\left[f\left(\hat{x},t\right)+g\left(\hat{x},t\right)u-\hat{\sigma }\right]+H\left(\varepsilon \right)\left(y-C\hat{x}\right)\\ \dot{\hat{\sigma }}=F\left(\varepsilon \right)\left(y-C\hat{x}\right) \end{array}\right.$$

where $\hat{x}$, $\hat{\sigma }={\left[\begin{array}{cc} {\hat{\sigma }}_{x} & {\hat{\sigma }}_{y} \end{array}\right]}^{T}$ are state variables and extended state variables, respectively.

(31) $$H\left(\varepsilon \right)=\left[\begin{array}{cc} h_{1} & 0\\ h_{2} & 0\\ 0 & h_{3}\\ 0 & h_{4} \end{array}\right]=\left[\begin{array}{cc} \frac{\alpha_{11}}{\varepsilon } & 0\\ \frac{\alpha_{21}}{\varepsilon^{2}} & 0\\ 0 & \frac{\alpha_{12}}{\varepsilon }\\ 0 & \frac{\alpha_{22}}{\varepsilon^{2}} \end{array}\right]\;F\left(\varepsilon \right)=\left[\begin{array}{cc} -h_{5} & 0\\ 0 & -h_{6} \end{array}\right]=\left[\begin{array}{cc} -\frac{\alpha_{31}}{\varepsilon^{3}} & 0\\ 0 & -\frac{\alpha_{32}}{\varepsilon^{3}} \end{array}\right]$$

where the role of the small design parameter ε>0 is: (1) adjusting the dynamic response speed of the observer; (2) Weakening the effects of nonlinear disturbance terms on observation accuracy. The parameters $\alpha_{ij},i=1,2,3,j=1,2$ are real numbers and should be chosen to satisfy the Hurwitz polynomials shown below:

(32) $$s^{3}+\alpha_{1j}s^{2}+\alpha_{2j}s+\alpha_{3j}\;j=1,2$$

### 4.2. EHGO Error Convergence Proof

We first build the singularly perturbed equation for the above GW nonlinear observer. For this, the observation error vector ***η*** can be defined as follows:

$$\eta ={\left[\begin{array}{cccccc} \eta_{1} & \eta_{2} & \eta_{{\hat{\sigma }}_{x}} & \eta_{3} & \eta_{4} & \eta_{{\hat{\sigma }}_{y}} \end{array}\right]}^{T}$$

where

(33) $$\eta_{1}=\frac{x_{1}-{\hat{x}}_{1}}{\varepsilon^{2}},\eta_{2}=\frac{x_{2}-{\hat{x}}_{2}}{\varepsilon },\eta_{3}=\frac{x_{3}-{\hat{x}}_{3}}{\varepsilon^{2}},\eta_{4}=\frac{x_{4}-{\hat{x}}_{4}}{\varepsilon }$$

(34) $$\eta_{\hat{\sigma }}=\left[\begin{array}{c} \eta_{{\hat{\sigma }}_{1}}\\ \eta_{{\hat{\sigma }}_{2}} \end{array}\right]=\hat{\sigma }+f\left(x,t\right)-f\left(\hat{x},t\right)+\left(g\left(x,t\right)-g\left(\hat{x},t\right)\right)u+w\left(x,t\right)$$

Combining Equations (29) and (30) with Equations (33) and (34), the singularly perturbed equation can be given by:

(35) $$\varepsilon \dot{\eta }=\bar{A}\eta +\varepsilon \bar{B}\left(\Delta_{x}+\Delta_{d}\right)$$

where

$$\bar{A}=\left[\begin{array}{cccccc} -\alpha_{11} & 1 & 0 & 0 & 0 & 0\\ -\alpha_{21} & 0 & 1 & 0 & 0 & 0\\ -\alpha_{31} & 0 & 0 & 0 & 0 & 0\\ 0 & 0 & 0 & -\alpha_{12} & 1 & 0\\ 0 & 0 & 0 & -\alpha_{22} & 0 & 1\\ 0 & 0 & 0 & -\alpha_{32} & 0 & 0 \end{array}\right]\;\bar{B}=\left[\begin{array}{cc} 0 & 0\\ 0 & 0\\ 1 & 0\\ 0 & 0\\ 0 & 0\\ 0 & 1 \end{array}\right]\;\begin{array}{c} \Delta_{x}=\dot{f}\left(x,t\right)-\dot{f}\left(\hat{x},t\right)\\ \Delta_{d}=\dot{w}\left(x,t\right)+\left(\dot{g}\left(x,t\right)-\dot{g}\left(\hat{x},t\right)\right)u \end{array}$$

Due to Assumption 1 and 2, there exists compact set $\Omega_{c}$ such that,

(36) $$x\left(t\right)\in \Omega_{c}\;for\;t\in \left[0,\infty \right)$$

Besides, considering that there is no singularity for the derivatives of f(x,t), $w(\omega_{b},x,t)$ and g(x,t) in Equation (29) and f(x,t) satisfies the Lipschitz condition in the closed interval of the state x, there exist constants $K_{x}>0,K_{d}>0$, such that $∥\Delta_{x}∥\leq K_{x}∥\eta ∥,∥\Delta_{d}∥\leq K_{d}$ for t∈[0,∞).

Defining Lyapunov function $V\left(\eta \right)$ and Lyapunov equation for singular perturbed Equation (35), respectively, as follows:

(37) $$V\left(\eta \right)=\varepsilon \eta^{T}P\eta$$

(38) $${\bar{A}}^{T}P+P\bar{A}=-Q$$

where P in Equation (37) is the unique positive definite solution of Equation (38), Q is an arbitrary positive-definite matrix, in particular, Q is given as identity matrix $I_{6\times 6}$. As previously mentioned, the parameters $\alpha_{ij},i=1,2,3,j=1,2$ in the matrix $\bar{A}$ are real and satisfy Hurwitz polynomial condition, so all the eigenvalues of the matrix $\bar{A}$ have negative real parts, which guarantees the existence of P. Taking the time derivative of V(η) along Equation (35), we have:

(39) $$\begin{array}{c} \dot{V}\left(\eta \right)=\eta^{T}\left(P\bar{A}+{\bar{A}}^{T}P\right)\eta +2\varepsilon \eta^{T}P\bar{B}\Delta_{x}+2\varepsilon \eta^{T}P\bar{B}\Delta_{d}\\ \;\leq -\left(1-2\varepsilon K_{x}∥P\bar{B}∥\right){∥\eta ∥}^{2}+2\varepsilon K_{d}∥P\bar{B}∥\cdot ∥\eta ∥ \end{array}$$

Obviously, there exists $\varepsilon^{*}>0$, such that $1-2\varepsilon K_{x}∥\mathrm{PB}∥>0$ for arbitrary $\varepsilon \in \left(0,\varepsilon^{*}\right)$.

Defining the auxiliary function as follow:

(40) $$l\left(\varepsilon \right)=\frac{2\varepsilon K_{d}∥\mathrm{PB}∥}{1-2\varepsilon K_{x}∥\mathrm{PB}∥}$$

Whenever $∥\eta ∥$ is such that $∥\eta ∥>l\left(\varepsilon \right)$, we have $\dot{V}\left(\eta \right)<0$. Therefore, there exists $T^{*}\left(\varepsilon \right)\in \left(0,\infty \right)$, such that the observation error vector η(t) enters the following set: $\Omega_{\eta }=\left\{\eta \in R^{6},∥\eta ∥\leq l\left(\varepsilon \right)\right\}$ and will remain in the set $\Omega_{\eta }$ in finite time $T^{*}\left(\varepsilon \right)$ for $\varepsilon \in \left(0,\varepsilon^{*}\right)$ and initial value η(0), which means:

(41) $$\eta \left(t\right)\in \Omega_{\eta },t\in \left[T^{*}\left(\varepsilon \right),\infty \right)$$

Therefore, the observation error converges to a small neighborhood of zero in finite time. Moreover, to meet the requirement of observation accuracy, it is supposed that the accuracy index is given by $∥\eta ∥\leq \delta_{\eta }$; we can find that the auxiliary function l(ε) is $O\left(\varepsilon \right)$ in the amplitude magnitude and is an increasing function of the design parameter *ε*. Hence, there exists $\varepsilon_{\eta }^{*}\leq \varepsilon^{*}$, such that, for every $\delta_{\eta }>0$ and arbitrary $\varepsilon \in \left(\left.0,\varepsilon_{\eta }^{*}\right]\right.$, the relationship $l\left(\varepsilon \right)\leq \delta_{\eta }$ is always true. Thus, the requirement of the observation error performance index $∥\eta ∥\leq \delta_{\eta }$ can be achieved in finite time.

According to the Equation (36), $g\left(x,t\right)-g\left(\hat{x},t\right)$ and $f\left(x,t\right)-f\left(\hat{x},t\right)$ in Equation (34) is Lipschitz and bounded, thus, the observation error index for the spacecraft angular rate related terms $w\left(x,t\right)$ can be transformed into the requirement on the observation error ***η***. Hence, the extended state $\hat{\sigma }$ can be regarded as the estimation of $w\left(x,t\right)$. According to the above analysis, we can conclude that there exists $\varepsilon_{w}^{*}\leq \varepsilon^{*}$ for any given $\delta_{w}>0$ such that, the Equation (42) is always true for every $\varepsilon \in \left(\left.0,\varepsilon_{w}^{*}\right]\right.$.

(42) $$∥\hat{\sigma }-w\left(x,t\right)∥\leq \delta_{w},t\in \left[T^{*}\left(\varepsilon \right),\infty \right]$$

From the above design process, the designed extended high gain observer can estimate the tilt angle accelerations of the rotor $\left({\dot{x}}_{2},{\dot{x}}_{4}\right)$ (that is, $\left({\ddot{\phi }}_{x},{\ddot{\phi }}_{y}\right)$) when implementing the estimation of the original state x. The tilt angle accelerations of the rotor $\left({\dot{x}}_{2},{\dot{x}}_{4}\right)$ mainly consist of two parts: the irrelevant part and the relevant part of the spacecraft angular rates $\omega_{b}$. The former can be regarded as the known model consisting of the GW inertia parameters, measurable variables and observable variables. Additionally, the latter is treated as an unknown factor due to the unknown spacecraft angular rates $\omega_{b}$. Thus, removing the known part, we can obtain the estimation of the other unknown related terms. Above all, the estimation of the spacecraft angular rates $\omega_{b}$ can be implemented indirectly by the estimation of tilt angular accelerations of rotor $\left({\dot{x}}_{2},{\dot{x}}_{4}\right)$ using the designed extended high gain observer with accuracy satisfied by adjusting the design parameter *ε*.

### 4.3. Influence Analysis of Measurement Noise

Theoretically, the observation accuracy can be obtained by decreasing the design parameter *ε*. However, in practice, the outputs of the tilt sensors of GW usually include measurement noise. Meanwhile, due to the differential characteristic of the above designed observer, a smaller design parameter *ε* will amplified the effects of measurement noise, which limits the range of the design parameter *ε* and affects the observation accuracy of the spacecraft angular rates $\omega_{b}$. The measurement equation with noise can be further given by:

(43) $$y=\mathrm{Cx}+n_{s}$$

where $n_{s}\in R^{2}$ is bounded measurement noise, which means that there exists positive number *μ* such that, the expression $∥n_{s}\left(t\right)∥\leq \mu$ holds. Then, the observation estimation error satisfies the following equation [16,17] given by:

(44) $$∥x\left(t\right)-\hat{x}\left(t\right)∥\leq \varepsilon c_{1}+\frac{\mu }{\varepsilon^{3}}c_{2}\overset{def}{=}F\left(\varepsilon ,\mu \right),\;\forall t\geq T$$

where $c_{1},c_{2}$ and *T* are positive constants.

For ε>0 and μ≥0, taking the partial derivatives of the function F(ε,μ) with respect to *ε*, we can obtain:

(45) $$\frac{\partial F(\varepsilon ,\mu )}{\partial \varepsilon }=c_{1}-\frac{3\mu c_{2}}{\varepsilon^{4}}$$

From Equation (45) we can find that: (1) F(ε,μ) is strictly decreasing for $\varepsilon <c_{a}\mu^{1/4}$, where $c_{a}={\left[3c_{2}/c_{1}\right]}^{1/4}$, strictly increasing for $\varepsilon >c_{a}\mu^{1/4}$, and has a global minimum $F_{min}\left(\varepsilon_{o},\mu \right)=\left(c_{1}c_{a}+c_{2}/c_{a}^{3}\right)\mu^{3}$ for $\varepsilon_{>}0$ at $\varepsilon_{o}=c_{a}\mu^{1/4}$; (2) For any given observation accuracy requirement $\delta_{w}$, $F_{min}<\delta_{w}$ for $\mu <{\left(\delta_{w}/k_{a}\right)}^{4}$, and the equation $F\left(\varepsilon ,\mu \right)=\delta_{w}$ has two solutions at $\varepsilon_{m}\leq \varepsilon_{o}$ and $\varepsilon_{M}>\varepsilon_{o}$: For $\varepsilon \leq \varepsilon_{o}$, we have the following relationship:

(46) $$F_{r}\left(\varepsilon_{m},\mu \right)=\varepsilon_{m}c_{1}+c_{2}\mu /\varepsilon_{m}^{3}=\delta_{w}\Rightarrow \varepsilon_{m}^{4}c_{1}+c_{2}\mu =\delta_{w}\epsilon_{m}^{3}\Rightarrow \varepsilon_{m}={\left(\frac{\mu c_{2}}{\delta_{w}\left(1-\frac{\varepsilon_{m}c_{1}}{\delta_{w}}\right)}\right)}^{\frac{1}{3}}$$

**Figure 4:**
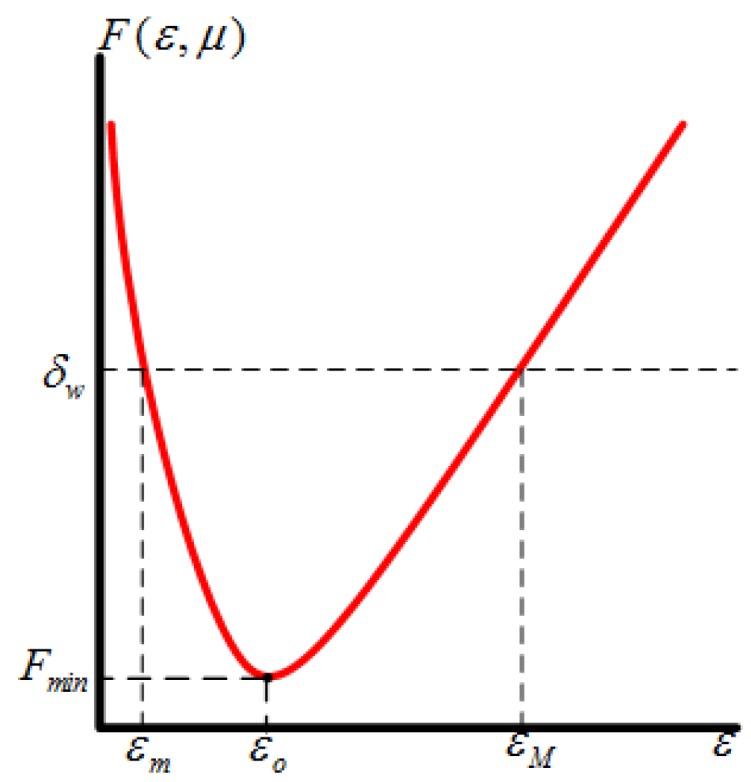
The changes of the extremum of estimation error.

Considering $\varepsilon \leq \varepsilon_{o}=c_{a}\mu^{1/4}$ and $\mu <{\left(\delta_{w}/k_{a}\right)}^{4}$, we have $\varepsilon <c_{a}\delta_{w}/k_{a}$, so that

(47) $$\varepsilon_{m}={\left(\frac{\mu c_{2}}{\delta_{w}\left(1-\frac{\varepsilon_{m}c_{1}}{\delta_{w}}\right)}\right)}^{\frac{1}{3}}\leq {\left(\frac{\mu c_{2}}{\delta_{w}\left(1-\frac{c_{1}c_{a}}{k_{a}}\right)}\right)}^{\frac{1}{3}}$$

where the equality happens only at μ=0. In addition, for $\varepsilon_{M}>\varepsilon_{o}$, since $\lim_{\mu \to 0}F\left(\varepsilon_{M},\mu \right)=\varepsilon_{M}c_{1}$, $\lim_{\mu \to 0}\varepsilon_{M}=\delta_{w}/c_{1}$. So we have $F_{r}\left(\varepsilon ,\mu \right)\leq \delta_{w}$ for all $\varepsilon \in (\varepsilon_{m},\varepsilon_{M}]$. According to the above analysis, the change sketch of the function F(ε,μ) as the parameter *ε* changing is shown in Figure 4.

From Equation (44) and Figure 4, we can find that due to the existence of measurement noise, the order of magnitude of the estimation error is $O(1/\varepsilon^{3})$, and too small or large *ε* will amplify the observation error; When $\varepsilon =\varepsilon_{o}=c_{a}\mu^{1/4}$, the minimum of the function $F_{min}\left(\varepsilon_{o},\mu \right)=\left(c_{1}c_{a}+c_{2}/c_{a}^{3}\right)\mu^{3}$ is achieved. Within a certain accuracy index $\delta_{w}$ the range of the design parameter *ε* is limited to $\left[\varepsilon_{m},\varepsilon_{M}\right]$. Considering the effect of the parameter *ε* on the observer performance recovery, a tradeoff should be considered between noise amplification and performance recovery including the state reconstruction speed [18].

## 5. Simulation

**Figure 5:**
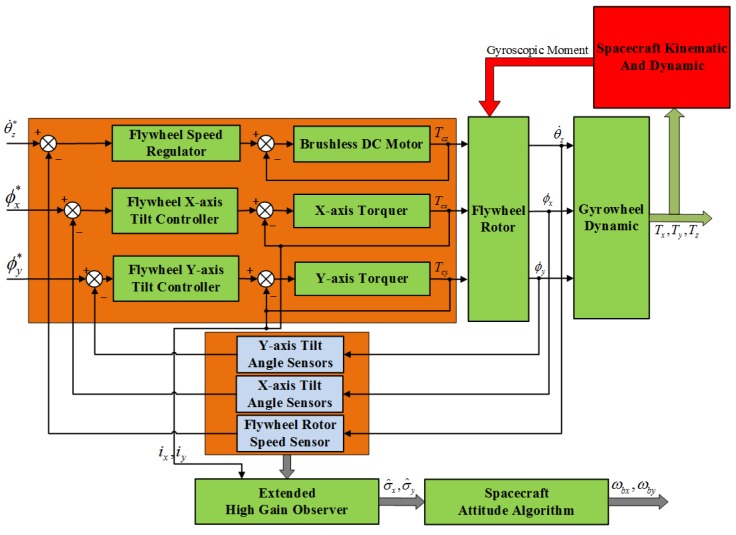
The changes of the extremum of estimation error.

In order to demonstrate the effectiveness of the proposed method, a simulation platform as Figure 5 is built. Utilizing this simulation platform, we will verify the performance of the extended high gain observer for estimating the spacecraft angular rates terms $w_{1}\left(\omega_{b},x,t\right)$ and $w_{2}\left(\omega_{b},x,t\right)$ in Equation (27).

In Figure 5, the variables ${\dot{\theta }}_{z}^{*},\phi_{x}^{*},\phi_{y}^{*}$ are the inputs of the motor control loop, the x-axis and the y-axis tilt control loop of the GW, respectively. The variables ${\dot{\theta }}_{z},\phi_{x},\phi_{y}$ are the corresponding measurable outputs of the above three control loops of the GW, respectively. The variables $T_{cz},T_{cx},T_{cy}$ are the control torque outputs of the above three control loops of the GW, respectively. The variables $i_{x},i_{y}$ are the measurable current outputs from the x-axis and the y-axis torquer, respectively. The relationship between $T_{cx},T_{cy}$ and $i_{x},i_{y}$ is given by $T_{cx}=k_{ty}i_{y},T_{cy}=k_{tx}i_{x}$, respectively. The variables $T_{z},T_{x},T_{y}$ acting on the spacecraft are the three-axis control torque outputs of the GW. The estimates ${\hat{\sigma }}_{x},{\hat{\sigma }}_{y}$ are the extended observer states, which represent the estimates of the related terms of the spacecraft angular motion in the GW dynamics equations along the x-axis and y-axis, respectively. The variables $\omega_{bx},\omega_{by}$ are the calculated values of spacecraft angular rates through the estimates ${\hat{\sigma }}_{x},{\hat{\sigma }}_{y}$ of the EHGO and the spacecraft attitude algorithm. According to the above description, the spacecraft attitude algorithm in Figure 5 can be expressed by:

(48) $$\begin{array}{cc} -{\hat{\sigma }}_{x}= & w_{1}\left(\omega_{b},x,t\right)\\ -{\hat{\sigma }}_{y}= & w_{2}\left(\omega_{b},x,t\right) \end{array}$$

where $w_{1}\left(\omega_{b},x,t\right)$ and $w_{2}\left(\omega_{b},x,t\right)$ are shown in Appendix A. Through solving the differential Equation (48), the spacecraft angular rates $\omega_{bx},\omega_{by}$ can be obtained in real time.

The key parameters in the simulation are given in the Table 1.

The estimation performance of the spacecraft angular rates with the above designed EHGO when GW outputting control torque $\left(T_{x},T_{y},T_{z}\right)$ should be validated, so the inputs of the two-dimensional tilt angles ($\phi_{x}^{*}$, $\phi_{y}^{*}$) and the motor speed (${\dot{\theta }}_{z}^{*}$) of GW can be given by:

$$\begin{array}{cc} \phi_{x}^{*} & =\phi_{y}^{*}=\left\{\begin{array}{c} 0.5\cdot t^{\circ }\;t\leq 10s\\ 0.1\cdot \sin{\left(2\pi \cdot 0.04\cdot t\right)}^{\circ }\;t>10s \end{array}\right.\\ {\dot{\theta }}_{z}^{*} & =157.04+22\cdot \sin\left(2\pi \cdot 0.02\cdot t\right)\;rad/s \end{array}$$

The initial values of spacecraft angular rates $\omega_{bx}$ and $\omega_{by}$ are set as 0.001 rad/s. Here, the design parameters of EHGO $\alpha_{ij}\;(i=1,2,3,j=1,2)$ in Equation (31) can be given by:

$$\alpha_{1j}=35.335,\;\alpha_{2j}=183.5681,\;\alpha_{3j}=705.5417,\;j=1,2$$

where the parameters $\alpha_{ij}\;(i=1,2,3,j=1,2)$ satisfy the Hurwitz polynomials as Equation (32), in addition, they are finally determined based on the design principle of control system to guarantee the dynamic response performance of EHGO.

To verify the effects of the design parameter *ε* of EHGO on the estimation accuracy of spacecraft angular rates ($\omega_{bx},\omega_{by}$) which is analyzed in Section 4.2, the design parameter *ε* is chosen as different positive real constants in simulation.

**Figure 6:**
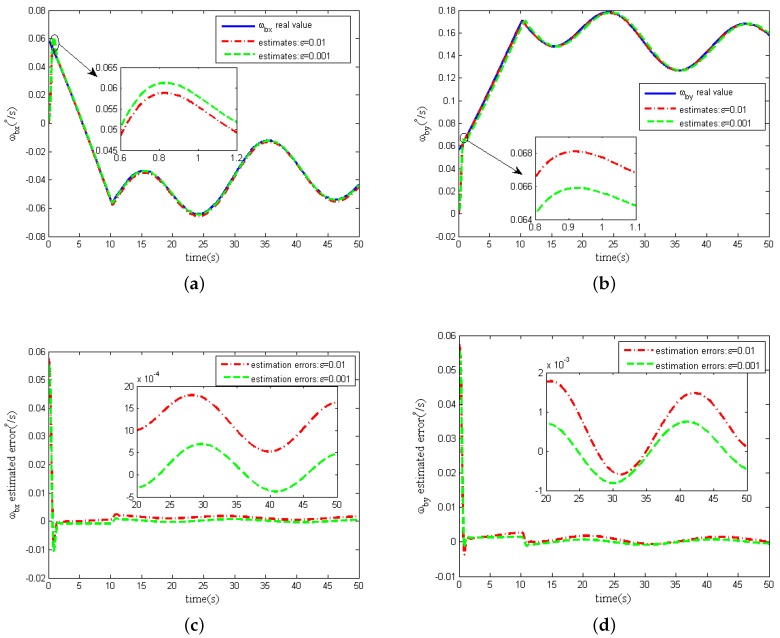
Estimation of spacecraft angular rates without measurement noise. (**a**) x-axis angular rate $\omega_{bx}$; (**b**) y-axis angular rate $\omega_{by}$; (**c**) $\omega_{bx}$ estimation error; (**d**) $\omega_{by}$ estimation error.

Without considering measurement noise in the two-dimensional tilt angle sensors, the estimates of the spacecraft angular rates ($\omega_{bx},\omega_{by}$) and estimation errors curves are shown in Figure 6.

From Figure 6a,b, we can see that when the design parameter *ε* is equal to both 0.01 and 0.001, the spacecraft angular rates ($\omega_{bx},\omega_{by}$) estimates can converge rapidly to the real spacecraft angular rates. The spacecraft angular estimation accuracy increases with the decrease of the value of *ε* from Figure 6c,d. Simulations with other values of the design parameter *ε* also show the same phenomenon. These phenomenons are also consistent with the error convergence proof in Section 4.2. The results for other values of *ε* are omitted in Figure 6.

Strictly, peaking effect of HGO will occur with the parameter *ε* decreasing [20], which means that the transient response of the estimates will change dramatically when the parameter *ε* becomes smaller. However, From the partial magnification of Figure 6a,b, the peaking effect is overcame by the saturation of control loop and the boundedness of the nominal model. Actually, for GW, the amplitude of the torque output from the two-dimensional torquers subject to the power limitation is always limited, which is set as less than 100 mNm in this paper, so the tilt control torques($T_{cx},T_{cy}$) acting on the rotor through tilt control loops are saturated and the peaking effect on the estimation performance of the extended state variables(${\hat{\sigma }}_{x},{\hat{\sigma }}_{y}$) can be naturally avoided. Besides, in the partial magnification of the Figure 6b, we can find that in the process of transient response the peaking values as ε=0.01 is larger than these values as ε=0.001, which is because the spacecraft angular rates $\omega_{bx},\omega_{by}$ is obtained indirectly by the estimates ${\hat{\sigma }}_{x},{\hat{\sigma }}_{y}$ in Equation (48).

**Figure 7:**
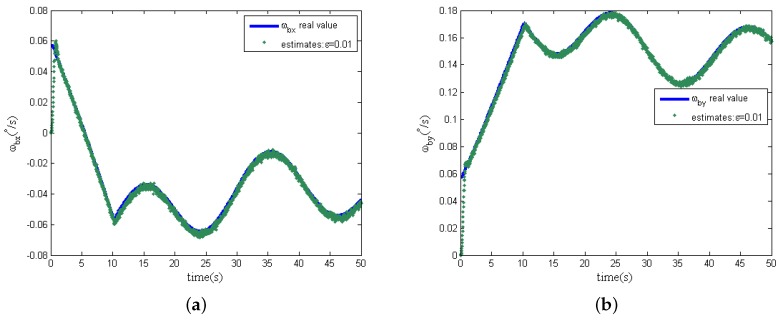
Estimation of spacecraft angular rates with measurement noise (ε=0.01). (**a**) x-axis angular rate $\omega_{bx}$; (**b**) y-axis angular rate $\omega_{by}$.

Further more, in the following simulation, measurement noise in the two dimensional tilt angle sensors is considered. The magnitudes of measurement noise in the tilt angle sensors are assumed to be uniformly distributed random variables taking values between $-0.5^{\circ }$ and $+0.5^{\circ }$, and the time interval of these random values variation is 0.0001s. In addition, the other simulation conditions remain unchanged, and the estimation curves of the spacecraft angular are shown in Figure 7a,b when the design parameter *ε* is chosen as 0.01 and 0.001, respectively.

**Figure 8:**
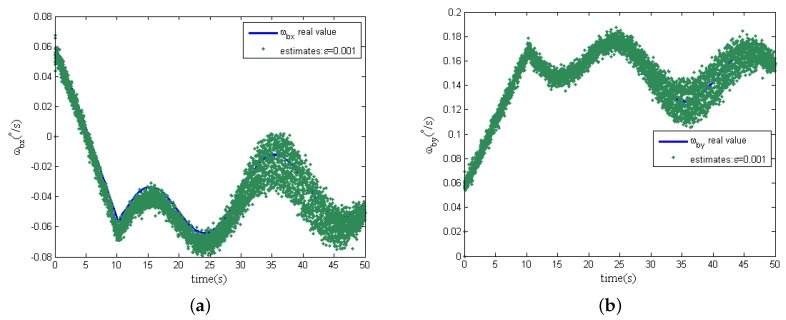
Estimation of spacecraft angular rates with measurement noise (ε=0.001). (**a**) x-axis angular rate $\omega_{bx}$; (**b**) y-axis angular rate $\omega_{by}$.

From Figure 7, when the design parameter *ε* is equal to 0.01, with the presence of measurement noise, the estimations of spacecraft angular rates can track the real angular rate instead of the deterioration of the estimation accuracy. However, differing from the previous simulation results without measurement noise, the design parameter *ε* is decreased to 0.001; the estimation noise is significantly amplified; and the estimation accuracy of the spacecraft angular rates becomes worse, as shown in Figure 8, because of the effect of measurement noise. Comparing Figure 7 and Figure 8, the phenomenon shows that the design parameter ε=0.01 is a comparatively reasonable tradeoff, as analyzed in Section 4.3 for the designed EHGO. It should be noted that the magnitudes of the measurement noise decide the optimal tradeoff of the design parameter *ε* between estimation performance improvement and noise amplification. Moreover, the statistics of measurement noise are not necessary for the high-gain observer design, which is different from the filtering approach, for which the imprecise knowledge of the measurement noise statistics seriously deteriorates the estimation, even resulting in instability. Although the motion of the spacecraft is unobservable from the sensors in the GW, the estimation of the spacecraft angular rates with the EHGO is independent of it. While for the filtering approach, the estimation of spacecraft angular rates [21] is improved by observability, and the observability [22] depends on the information of the spacecraft dynamical model.

## 6. Conclusions

In this paper, the estimation of the spacecraft rates with GW based on extended high gain observer, which works in large tilt angles for the radial torque output, add, is first proposed and studied. For this purpose, three major contributions of this paper can be summarized as follows:

(1) A complete dynamical model of GW is built with chosen generalized coordinates ($\theta_{x},\theta_{y},\theta_{z}$) by Lagrange’s Method, and since the generalized coordinates ($\theta_{x},\theta_{y}$) and its derivatives in the GW dynamical model are unmeasurable, the relationships between the unmeasurable generalized coordinates and the measurable variables ($\phi_{x},\phi_{y},{\dot{\theta }}_{z},\theta_{z}$) by sensors are derived to construct the nonlinear state equation expressed by measurable variables for the spacecraft rate estimations with the GW.
(2) The affine nonlinear state equation of GW and measurement equation are built based on the contribution (1). Combining the affine nonlinear state equation with measurement equation and extending the relevant terms of spacecraft angular rates as states, a high gain observer is designed to estimate the relevant terms of the spacecraft angular rates. Through solving the known differential equation, the spacecraft angular rates can be calculated.
(3) The stability of the designed EHGO in contribution (2) is proved by Lyapunov’s stability theory, and the effects of the design parameter *ε* and measurement noise on the estimation accuracy are also analyzed.

## Figures and Tables

**Table 1:** Gyrowheel and Spacecraft Design Parameter in Simulation [19].

Parameter Name	Value
Rotor transverse-axis inertia $I_{rt}$	$3.458\times 10^{-3}$ kg · m^2^
Rotor spin-axis inertia $I_{rs}$	$6.402\times 10^{-3}$ kg · m^2^
Gimbal transverse-axis inertia $I_{gt}$	$1.2758\times 10^{-5}$ kg · m^2^
Gimbal spin-axis inertia $I_{gs}$	$1.8047\times 10^{-5}$ kg · m^2^
Torsion Spring Stiffness $k_{x},k_{y}$	0.092Nm/rad
Torsion Spring damping $c_{x},c_{y}$	0 Nm/(rad/s)
Spacecraft Inertia $I_{\mathrm{sat}}$	$diag\left[\begin{array}{ccc} 50 & 50 & 50 \end{array}\right]$ kg · m^2^

## Abbreviations

The following abbreviations are used in this manuscript:

GW7	Gyrowheel
ACS	Attitude control system
HGO	High gain observer
EHGO	Extended high gain observer
CMG	Control moment gyroscope
MSDGCMG	Magnetically suspended double-gimbal control moment gyroscope
AMBs	Active magnetic bearings
DOF	Degrees of freedom
SINS	Strapdown inertial navigation system
DTG	Dynamically tuned gyroscope
VSDGCMG	Variable speed control moment gyroscope

## Appendix A

$$\begin{array}{c} \begin{array}{cc} f_{1}\left(x,t\right)= & \frac{{\dot{\phi }}_{y}S_{\phi_{y}}}{C_{\phi_{y}}^{2}}\left[C_{\phi_{z}}C_{\theta_{y}}{\dot{\theta }}_{x}-S_{\phi_{z}}{\dot{\theta }}_{y}-\left(C_{\theta_{x}}S_{\theta_{y}}C_{\phi_{z}}+S_{\theta_{x}}S_{\phi_{z}}\right){\dot{\theta }}_{z}\right]+\\ & \frac{1}{C_{\phi_{y}}}\left[\begin{array}{cc} & \left(-{\dot{\phi }}_{z}S_{\phi_{z}}C_{\theta_{y}}-{\dot{\theta }}_{y}C_{\phi_{z}}S_{\theta_{y}}\right){\dot{\theta }}_{x}-{\dot{\phi }}_{z}C_{\phi_{z}}{\dot{\theta }}_{y}-\left(C_{\theta_{x}}S_{\theta_{y}}C_{\phi_{z}}+S_{\theta_{x}}S_{\phi_{z}}\right){\ddot{\theta }}_{z}+\\ & \left({\dot{\phi }}_{z}C_{\theta_{x}}S_{\theta_{y}}S_{\phi_{z}}-C_{\phi_{z}}\left({\dot{\theta }}_{y}C_{\theta_{x}}C_{\theta_{y}}-{\dot{\theta }}_{x}S_{\theta_{x}}S_{\theta_{y}}\right)-{\dot{\phi }}_{z}S_{\theta_{x}}C_{\phi_{z}}-C_{\theta_{x}}S_{\phi_{z}}{\dot{\theta }}_{x}\right){\dot{\theta }}_{z} \end{array}\right]-\\ & \frac{\beta_{1}}{2I_{1}}\left(2c_{x}{\dot{\theta }}_{x}+2k_{x}\theta_{x}+I_{2}S_{2\theta_{x}}{\dot{\theta }}_{z}^{2}+2I_{3}S_{2\theta_{y}}{\dot{\theta }}_{x}{\dot{\theta }}_{y}+2\left(I_{3}C_{2\theta_{y}}-I_{ry}\right)C_{\theta_{x}}{\dot{\theta }}_{y}{\dot{\theta }}_{z}\right)-\\ & \frac{\eta }{2I_{ry}}\left(-2c_{y}{\dot{\theta }}_{y}-2k_{y}\theta_{y}-I_{3}C_{\theta_{x}}^{2}S_{2\theta_{y}}{\dot{\theta }}_{z}^{2}+I_{3}S_{2\theta_{y}}{\dot{\theta }}_{x}^{2}+2\left(I_{3}C_{2\theta_{y}}-I_{ry}\right)C_{\theta_{x}}{\dot{\theta }}_{x}{\dot{\theta }}_{z}\right)\\ f_{2}\left(x,t\right)= & \left({\dot{\phi }}_{z}C_{\theta_{y}}C_{\phi_{z}}-{\dot{\theta }}_{y}S_{\theta_{y}}S_{\phi_{z}}\right){\dot{\theta }}_{x}-S_{\phi_{z}}{\dot{\phi }}_{z}{\dot{\theta }}_{y}-\left(C_{\theta_{x}}S_{\theta_{y}}S_{\phi_{z}}-S_{\theta_{x}}C_{\phi_{z}}\right){\ddot{\theta }}_{z}-\\ & \left[{\dot{\phi }}_{z}C_{\theta_{x}}S_{\theta_{y}}C_{\phi_{z}}+S_{\phi_{z}}\left({\dot{\theta }}_{y}C_{\theta_{x}}C_{\theta_{y}}-{\dot{\theta }}_{x}S_{\theta_{x}}S_{\theta_{y}}\right)-\left({\dot{\theta }}_{x}C_{\theta_{x}}C_{\phi_{z}}-{\dot{\phi }}_{z}S_{\theta_{x}}S_{\phi_{z}}\right)\right]{\dot{\theta }}_{z}+\\ & \frac{\beta_{2}}{2I_{1}}\left(-2c_{x}{\dot{\theta }}_{x}-2k_{x}\theta_{x}-I_{2}S_{2\theta_{x}}{\dot{\theta }}_{z}^{2}-2I_{3}S_{2\theta_{y}}{\dot{\theta }}_{x}{\dot{\theta }}_{y}-2\left(I_{3}C_{2\theta_{y}}-I_{ry}\right)C_{\theta_{x}}{\dot{\theta }}_{y}{\dot{\theta }}_{z}\right)+\\ & \frac{C_{\phi_{z}}}{2I_{ry}}\left(-2c_{y}{\dot{\theta }}_{y}-2k_{y}\theta_{y}-I_{3}C_{\theta_{x}}^{2}S_{2\theta_{y}}{\dot{\theta }}_{z}^{2}+I_{3}S_{2\theta_{y}}{\dot{\theta }}_{x}^{2}+2\left(I_{3}C_{2\theta_{y}}-I_{ry}\right)C_{\theta_{x}}{\dot{\theta }}_{x}{\dot{\theta }}_{z}\right) \end{array} \end{array}$$

$$\begin{array}{c} \begin{array}{cc} w_{1}\left(\omega_{b},x,t\right)= & \left(\beta_{1}B_{1}\left(\theta \right)-\eta D_{1}\left(\theta \right)\right)\omega_{bx}+\left(\beta_{1}B_{2}\left(\theta \right)-\eta D_{2}\left(\theta \right)\right)\omega_{by}+\\ & \left(\beta_{1}B_{3}\left(\theta \right)-\eta D_{3}\left(\theta \right)\right){\dot{\omega }}_{bx}+\left(\beta_{1}B_{4}\left(\theta \right)-\eta D_{4}\left(\theta \right)\right){\dot{\omega }}_{by}+\\ & \left(\beta_{1}B_{5}\left(\theta \right)-\eta D_{5}\left(\theta \right)\right)\omega_{bx}^{2}+\left(\beta_{1}B_{6}\left(\theta \right)-\eta D_{6}\left(\theta \right)\right)\omega_{by}^{2}+\left(\beta_{1}B_{7}\left(\theta \right)-\eta D_{7}\left(\theta \right)\right)\omega_{bx}\omega_{by}\\ w_{2}\left(\omega_{b},x,t\right)= & \left(\beta_{2}B_{1}\left(\theta \right)-C_{\phi_{z}}D_{1}\left(\theta \right)\right)\omega_{bx}+\left(\beta_{2}B_{2}\left(\theta \right)-C_{\phi_{z}}D_{2}\left(\theta \right)\right)\omega_{by}+\\ & \left(\beta_{2}B_{3}\left(\theta \right)-C_{\phi_{z}}D_{3}\left(\theta \right)\right){\dot{\omega }}_{bx}+\left(\beta_{2}B_{4}\left(\theta \right)-C_{\phi_{z}}D_{4}\left(\theta \right)\right){\dot{\omega }}_{by}+\\ & \left(\beta_{2}B_{5}\left(\theta \right)-C_{\phi_{z}}D_{5}\left(\theta \right)\right)\omega_{bx}^{2}+\left(\beta_{2}B_{6}\left(\theta \right)-C_{\phi_{z}}D_{6}\left(\theta \right)\right)\omega_{by}^{2}+\left(\beta_{2}B_{7}\left(\theta \right)-C_{\phi_{z}}D_{7}\left(\theta \right)\right)\omega_{bx}\omega_{by} \end{array}\\ \begin{array}{cc} g_{x}^{1}\left(x,t\right)= & \frac{\beta_{1}}{I_{1}}C_{\theta_{z}}+\frac{\eta }{I_{ry}}S_{\theta_{z}}C_{\theta_{x}}\;g_{y}^{1}\left(x,t\right)=\frac{\beta_{1}}{I_{1}}S_{\theta_{z}}-\frac{\eta }{I_{ry}}C_{\theta_{z}}C_{\theta_{x}}\\ g_{x}^{2}\left(x,t\right)= & \frac{\beta_{2}}{I_{1}}C_{\theta_{z}}-\frac{C_{\phi_{z}}}{I_{ry}}S_{\theta_{z}}C_{\theta_{x}}\;g_{y}^{2}\left(x,t\right)=\frac{\beta_{2}}{I_{1}}S_{\theta_{z}}+\frac{C_{\phi_{z}}}{I_{ry}}C_{\theta_{z}}C_{\theta_{x}} \end{array} \end{array}$$

where, $B_{i}\left(\theta \right),D_{i}\left(\theta \right),i=1,2,...,7$ has been shown in Equations (16) and (17), and the unmeasurable variables ($\phi_{z},\theta_{x},\theta_{y},{\dot{\theta }}_{x},{\dot{\theta }}_{y}$) in the above equations are substituted by following equations:

$$\begin{array}{c} \begin{array}{cc} \beta_{1}= & \frac{C_{\phi_{z}}C_{\theta_{y}}}{I_{1}C_{x_{3}}}\;\eta =\frac{S_{\phi_{z}}}{I_{ry}C_{x_{3}}}\;\beta_{2}=\frac{C_{\theta_{y}}S_{\phi_{z}}}{I_{1}}\\ \theta_{x}= & arcsin\left(C_{x_{1}}S_{x_{3}}S_{\phi_{z}}+S_{x_{1}}C_{\phi_{z}}\right)\;\theta_{y}=arcsin\left(S_{x_{3}}C_{\theta_{z}}-S_{x_{1}}C_{x_{3}}S_{\theta_{z}}\right)\\ {\dot{\theta }}_{x}= & \frac{1}{C_{\theta_{y}}}\left(C_{x_{3}}C_{\phi_{z}}x_{2}+S_{\phi_{z}}x_{4}+C_{\theta_{x}}S_{\theta_{y}}{\dot{\theta }}_{z}\right)\;{\dot{\theta }}_{y}=C_{\phi_{z}}x_{4}-C_{x_{3}}S_{\phi_{z}}x_{2}-S_{\theta_{x}}{\dot{\theta }}_{z} \end{array} \end{array}$$

where $\phi_{z}=arctan\frac{C_{x_{1}}S_{\theta_{z}}}{C_{x_{3}}C_{\theta_{z}}+S_{x_{1}}S_{x_{3}}S_{\theta_{z}}}$.
